# Engineering More Stable, Selectable Marker-Free Autoluminescent Mycobacteria by One Step

**DOI:** 10.1371/journal.pone.0119341

**Published:** 2015-03-11

**Authors:** Feng Yang, Moses M. Njire, Jia Liu, Tian Wu, Bangxing Wang, Tianzhou Liu, Yuanyuan Cao, Zhiyong Liu, Junting Wan, Zhengchao Tu, Yaoju Tan, Shouyong Tan, Tianyu Zhang

**Affiliations:** 1 State Key Laboratory of Respiratory Disease, Guangzhou Institutes of Biomedicine and Health, Chinese Academy of Sciences, Guangzhou, Guangdong, China; 2 State Key Laboratory of Respiratory Disease, Department of Clinical Laboratory, The Guangzhou Chest Hospital, Guangzhou, Guangdong, China; University of Padova, Medical School, ITALY

## Abstract

In our previous study, we demonstrated that the use of the autoluminescent *Mycobacterium tuberculosis* as a reporter strain had the potential to drastically reduce the time, effort, animals and costs consumed in evaluation of the activities of drugs and vaccines in live mice. However, the strains were relatively unstable and lost reporter with time without selection. The kanamycin selection marker used wasn’t the best choice as it provides resistance to amino glycosides which are an important class of second line drugs used in tuberculosis treatment. In addition, the marker could limit utility of the strains for screening of new potential drugs or evaluating drug combinations for tuberculosis treatment. Limited selection marker genes for mycobacterial genetic manipulation is a major drawback for such a marker-containing strain in many research fields. Therefore, selectable marker-free, more stable autoluminescent mycobacteria are highly needed. After trying several strategies, we created such mycobacterial strains successfully by using an integrative vector and removing both the resistance maker and integrase genes by Xer site-specific recombination in one step. The corresponding plasmid vectors developed in this study could be very convenient in constructing other selectable marker-free, more stable reporter mycobacteria with diverse applications.

## Introduction

Many severe bacterial diseases, such as tuberculosis (TB), leprosy and Buruli ulcers are caused by mycobacteria. For example, TB, an infectious disease caused by *Mycobacterium tuberculosis* (MTB), is one of the greatest single infectious diseases causing morbidity and death in the world. The only TB vaccine in use for over 90 years, *Mycobacterium bovis* BCG (BCG), has very limited protection efficacy in older children and adults. The 9.0 million incident cases of TB, 1.5 million deaths from TB patients in 2013 alone [1], and the appearance of multi drug-resistant (MDR) [2,3], extensively drug-resistant (XDR) [2,3] and even totally drug-resistant (TDR) TB [4] presents a striking reminder of the magnitude of destruction caused by TB. All these indicate that new, more effective drugs and vaccines are urgently needed.

Routine drug susceptibility testing for MTB depends on a positive culture for diagnosis after which a drug susceptibility test is performed which usually takes 3–6 weeks [5]. The slow diagnosis and in some cases inaccurate or false negative phenotypic results [6], is a major contributor to the current drug resistant epidemic and hindrance to mycobacterial research. The Buruli ulcers causing pathogen, *Mycobacterium ulcerans*, grows even much slower as 3 months are needed for counting the visible colonies after plating. The necessity to work under stringent biosafety level 3-containment also makes studies of MTB very expensive, especially for long-term use facilities. Therefore, the lack of an effective, rapid, reliable and inexpensive reporter strain in TB research, especially for *in vivo* studies, is a major drawback.

In our previous studies [7,8], we constructed autoluminescent MTB and *Mycobacterium ulcerans* as reporter strains which expressed the *luxCDABE* operon from *Photorhabdus luminescens* [9]. The operon encodes enzymes for both light production and for recycling reaction substrates. Therefore, use of the autoluminescent reporter strains for testing drugs does not need the addition of an exogenous substrate. The same samples can be monitored in real time, and the colony forming units (CFU) and the light intensity (relative light unit, RLUs) correlate very well. Use of this system demonstrates the potential to drastically reduce the time, effort, animals and costs consumed in evaluation of the activities of drugs and vaccines in live mice as it only takes 3 seconds to detect light in live mouse using an inexpensive device [7,8]. The autoluminescent strains created have been proved to be essentially as virulent as their wild-type parent strains and the drug susceptibilities including for aminoglycosides such as streptomycin are not affected except for kanamycin (KAN) which was used as a selection marker. These properties make the reporter strains appealing for testing drug activity both *in vitro* and *in vivo* as only very small amount of samples, a few mice and short time are needed to infer the activity of a compound with very good reproducibility and no addition of exogenous substrate. However, the strains are relatively unstable probably as a result of excision of the *luxCDABE* operon by the L5 mycobacteriophage integrase at a very low rate [10,7]. This assumption was recently approved in a similar study describing the construction of a recombinant MTB expressing firefly luciferase gene in an integrative plasmid with the integrase gene removed [11]. In addition, the selection marker used could have been inappropriate for molecular genetic manipulation [12], screening of potential drug combinations and testing therapeutic regimens containing KAN *in vivo* due to possible cross drug resistance. The limited antibiotic resistance markers for mycobacterial genetic manipulation pose a serious challenge, and therefore, development of selectable marker-free, more stable, autoluminescent mycobacteria is highly needful.

The strategies for construction of selectable marker-free mycobacterial strains are summarized in our recently published report [12]. Herein, we tried 2 main strategies for constructing selectable marker-free mycobacteria. The antibiotic resistance cassette flanked by two short DNA sequences in direct orientation could possibly be recognized and removed either by the exogenous resolvase or the endogenous mycobacterial recombinases XerCD. The integrase gene also needed to be removed to make the autoluminescent mycobacteria more stable. We finally succeeded using an integrative plasmid expressing the natural *luxCDABE* operon from *Photorhabdus luminescens* [9] at the downstream of *Hsp60* promoter [13]. The L5 integrase gene (*int*) and hygromycin (HYG)-resistant gene in the same cassette were resolved by the endogenous XerC and XerD recombinases [14] using our recently published system [12]. The selectable marker-free strains were proved to be more stable than the previously reported ones and could be widely used in anti-mycobacterial drug screening and evaluation. Additionally, their derivative strains could possibly be used widely in many research fields of mycobacteria.

## Materials and Methods

### Bacterial strains (Table 1) and culture media

*Escherichia coli* strain DH5α [15] and the corresponding transformants were grown at 37°C in Luria-Bertani (LB) broth or on agar containing KAN (Invitrogen), ampicillin (Sigma-Aldrich, USA) or HYG (Roche Diagnostics, Switzerland) at final concentrations (μg/ml) of 40, 100 and 200, respectively. MTB H37Rv [16], MTB H37Ra [17] and *Mycobacterium bovis* BCG Tice (BCG) [18,19] were grown at 37°C in Middlebrook 7H9 broth (Becton Dickinson, USA) supplemented with 10% oleic acid albumin dextrose catalase (OADC, Becton Dickinson, USA) and 0.05% Tween80 where indicated, or on 7H11 agar supplemented with OADC. *M*. *smegmatis* mc^2^155 (MSM) [20] was grown in LB broth or on LB agar or Middlebrook 7H11 agar (Difco) supplemented with albumin dextrose catalase at 37°C. KAN, HYG, carbenicillin and cycloheximide were added to agar when required to final concentrations (μg/ml) of 40, 50, 50 and 10 respectively for MTB and BCG, and the same concentrations for MSM except for HYG 150. The concentrations (μg/ml) in liquid broth were KAN 20 and HYG 100 for MSM and HYG 10 for MTB and BCG.

**Table 1:** Bacterial strains in this study.

Strains	Relevant characteristic(s)	Source or reference
*E*. *coli* DH5α	General-purpose cloning strain; F^-^ [φ80d *lacZΔM15*] *Δ*D(*lacZYA-argF*)*U169 deoR recA1 endA1 hsdR17 glnV44 thi-1 gyrA96 relA*	[11]
*M*. *smegmatis* mc^2^155	Highly transformable derivative of ATCC 607	[16]
MSM-OHP	MSM cotransformed with pOHP and pInt	This study
MSM-OHP	MSM cotransformed with pOHP and pInt	This study
AlMSMT1	MSM containing pOHIhd	This study
AlMSMT2	MSM containing pOPHI	This study
UAlMSM	Selectable marker-free autoluminescent MSM	This study
*M*. *tuberculosis* H37Rv	Widely used virulent laboratory MTB strain, ATCC 27294	[12]
AlRv	Autoluminescent MTB H37Rv resistant to KAN	[7]
AlRvT1	MTB H37Rv::pOHIhd, MTB H37Rv containing pOHIhd	This study
AlRvT2	MTB H37Rv::pOPHI MTB, H37Rv containing pOPHI	This study
UAlRv	Selectable marker-free autoluminescent MTB H37Rv	This study
*M*. *tuberculosis* H37Ra	Widely used avirulent laboratory MTB strain, ATCC25177	[13]
AlRaT2	MTB H37Ra::pOPHI MTB, H37Ra containing pOPHI	This study
UAlRa	Selectable marker-free autoluminescent MTB H37Ra	This study
*M*. *bovis* BCG Tice	The live attenuated TB vaccine	[15]
AlBCGT2	BCG::pOPHI, BCG containing pOPHI	This study
UABCG	Selectable marker-free autoluminescent BCG	This study

ATCC: The American Type Culture Collection.

### General DNA techniques

For polymerase chain reaction (PCR) amplification reactions were performed with *pfu* DNA polymerase (Takara) and 5% DMSO was added due to the high G+C content of the mycobacterial genomes. The PCR products were analyzed by electrophoresis in agarose gels and purified using a DNA gel extraction kit (Bioflux). Plasmids were also extracted and purified using kits from the same company. Purified PCR products, plasmids or plasmids transformed into *E*. *coli* strains were sequenced at BGI, Shenzhen, China. MSM was transformed as previously described [20], while MTB and BCG were transformed as previously described [21] with some modifications. The competent MTB and BCG cells were first incubated at 37°C for 10 min before electroporation, and transformation was performed at room temperature. The genomic mycobacterial DNA was extracted using the CTAB method as previously described [15].

### Construction of marker-free autoluminescent mycobacteria using the endogenous Xer recombinase system

**Fig 1:**
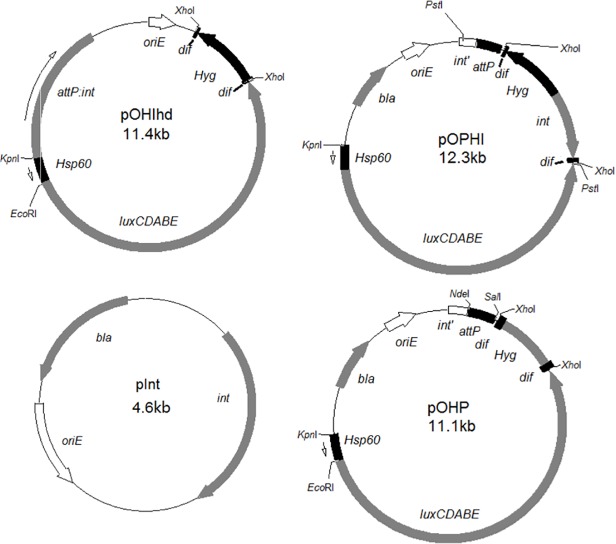
The plasmids constructed in this study for transforming into mycobacteria to create unmarked autoluminescent mycobacteria. *oriE*, origin region of *E*. *coli*; *Hsp60*, the strong mycobacterial promoter; *luxCDABE*, the operon for producing autoluminescence; *bla*, ampicillin resistance gene; *Kan*, KAN resistance gene; *res*, the transposonγδ resolvase action site; *attP*, mycobacteriophage L5 attachment site; *int*, integrase gene; *int’*, the remaining part of integrase gene; *attB*, attachment site from the mycobacterial genome corresponding to *attP*; *oriM*, origin region of mycobacteria; *Hyg*, HYG resistance gene; *dif*, the recombinases XerCD action site.

This strategy was designed to deliver the *Hsp60-luxCDABE* into the mycobacterial genome by an integrative plasmid. The *Hyg* gene could then either be removed by the endogenous Xer recombinase system to form selectable marker-free strains (plasmid pOHIhd, Table 2, Fig. 1), or alternatively, both *Hyg*+*int* genes could be removed together by the same system to form selectable marker-free and more stable strains (plasmid pOPHI, Table 2, Fig. 1). In addition, we co-transformed the suicide plasmid pInt containing the *int* gene (Fig. 1, Table 2) with plasmid pOHP containing the *attP* site, the *luxCDABE* (under the regulation of the strong *Hsp60* promoter, *Hsp60-luxCDABE*) [15], and the *dif*-ΩHYG-*dif* cassette (Fig. 1, Table 2).

**Table 2:** Plasmids in this study.

The plasmids	Relevant characteristic(s)	Source or reference
pInt	*int*, AMP^r^, *ori E*, can not multiply in mycobacteria	Fig. 1.
pblueInt	The pbluescript SK(+) inserted with *attP*:*int* from pMH94	S3 Fig. [7]
pTYP	*attP*, AMP^r^, *ori E*, can not multiply in mycobacteria	S3 Fig.
pluxOK	*Hsp60-luxCDABE*, *ori E*, KAN^r^, AMP^r^	[7]
pTYOP	*Hsp60-luxCDABE*, *attP*, AMP^r^, *ori E*, can not multiply in mycobacteria	S2 and S3 Figs
pUC19	AMP^r^, *ori E*, general-purpose cloning vector	S1 Fig.
pTYdHm	pUC19 containing *dif*-ΩHYG-*dif* at *Kpn*I-*Hin*dIII sites	[12]
pdH3	pUC19 containing *dif*-ΩHYG-*dif* at *Hin*dIII site	S1 Fig.
pTYOHd	pdH3 inserted with *Hsp60-luxCDABE*	S1 Fig.
pOHIhd	pTYOHd inserted with *attP*:*int*	Fig. 1, S1 Fig.
pTYd	pUC19 containing *dif*-*dif* at *Kpn*I-*Hin*dIII sites	S2 Fig. [12]
pTYdI	pTYd inserted with *int* in between *dif*-*dif*	S2 Fig.
pTYdIH	pUC19 containing *dif*-*Hyg*-*int*-*dif*	S2 Fig.
pOPHI	pTYOP inserted with *dif*-*Hyg*-*int*-*dif*	Fig. 1, S2 Fig.
pOHP	pTYOP inserted with *dif*-ΩHYG-*dif*	Fig. 1
pBluescript II SK(+)	AMP^r^, *ori E*, general-purpose cloning vector	[7]
pBlueI	Derived from pInt for giving *int*	S2 Fig.

AMP: ampicillin; KAN: kanamycin; HYG: hygromycin.

To construct plasmid pOHIhd, the *dif*-ΩHYG-*dif* cassette was excised with *Hin*dIII from plasmid pTYdHm (Table 2) [12] and inserted into plasmid pUC19 (Table 2) digested with the same enzyme to form pdH3 (Table 2, S1 Fig.). The *Kpn*I-*Hsp60-luxCDABE*-*Pst*I was then excised from pluxOK (Table 2) [7] and inserted into pdH3 (Table 2) digested with the same enzymes to form pTYOHd (S1 Fig., Table 2). The *attP*:*Int* excised with *Kpn*I and *Sma*I from pblueInt (S1 Fig., Table 2) [7] was then inserted into pTYOHd digested with *Kpn*I and *Sca*I to form pOHIhd (S1 Fig. and Fig. 1, Table 2). To construct the plasmid pOPHI (Table 2), the *int* gene, amplified with primers Intf and Intr (Table 3) from the plasmid pblueInt (Table 2), was excised with *Xba*I and *Cla*I, inserted into the common plasmid pBluescript II SK(+) to form pBlueI (Table 2), and then sequenced. The *int* gene in pBlueI was then excised with *Xba*I and *Cla*I and inserted into pTYd [12] to form pTYdI (S2 Fig., Table 2). The optimized *Hyg* gene [12] in pTYdHm (Table 2) was cut with *Xba*I and then inserted into the *Xba*I site of pTYdI to form pTYdIH (S2 Fig., Table 2). The direction of the *Hyg* gene was verified by restriction mapping analysis. The *dif*-ΩHYG-*int*-*dif* cassette was then excised from pTYdIH and inserted into pTYOP to form pOPHI (S2 Fig. and Fig. 1, Table 2). To construct plasmid pTYOP, the plasmid pblueInt was digested with *Pst*I and self-ligated to form pTYP (S3 Fig., Table 2) with *int* gene removed. The *Hsp60-luxCDABE* excised from pluxOK (Table 2) [7] with *Kpn*I-*Xho*I was inserted into pTYP to give pTYOP (S3 Fig.).The *dif*-ΩHYG-*dif* cassette was excised from the plasmid pTYd constructed in our previous work (Table 2) [12] with *Xho*I and inserted into the same site of pTYOP to give pOHP (Fig. 1, Table 2).

**Table 3:** DNA primers used in this study.

Primer pairs	The function of the primers	Nucleotide sequence (5'-3') with enzyme sites underlined (forward primer/reverse primer)
Intf/ Intr	Flanking the *int* gene for cloning it without the *attP* site.	GCTCTAGACTAGTTTGGAAGAATGGGTGTCT/CCATCGATCTCAGTGTCCTTGGGAGGG
Hyg0702-f/Hyg0702-r	Corresponding to an inner part of *Hyg* for detecting existence of this gene.	AGAGCACCAACCCCGTACTG/GTGAAGTCGACGATCCCGGT
Int0702-f/Int0702-r	Corresponding to an inner part of *Int* for detecting existence of this gene.	TTCATGTGCGCTCGGATCAT/TCACGCTGGAGGAGTACACC
noHI-f/noHI-r	Flanking *Int-Hyg* in the plasmid pOPHI for detecting existence of these 2 genes.	TGGATGCGTCAGCAACCAGT/ CAGAGATGGTGCCCTTGGTG
attB1210-f/ attB1210-r	MTB for verifying if the plasmid integrated was dissociated from the genome.	CCTGTTTGGCCAGCTCTTTG/TGCCTTGGTACCGGACAGCA
luxAB-f/luxAB-r	Corresponding to an inner part of *luxAB* for detecting existence of these genes or the *luxCDABE* operon.	GGTTTATGTGGTGGCTGAAT/GCCGACAACACCATTATCTG

### Construction and verification of the target autoluminescent strains

Mycobacteria transformed with pOHIhd or pOPHI or co-transformed with pInt and pOHP (Table 2) were selected on HYG-containing plates. The autoluminescent mycobacterial colonies with deleted HYG-resistant gene were selected by streaking them in the presence and absence of HYG after several passages in plain 7H9 broth and further tested by PCR using appropriate primers (Table 3).The primer pair Hyg0702-f and Hyg0702-r was for testing the loss of the *Hyg* gene; Int0702-f and Int0702-r for testing the loss of the *int* gene; while noHI-f (corresponding to 170 bp from the end of *luxE* gene) and noHI-r (corresponding to the end of *attP* near *luxCDABE* in plasmid pTYOP) was for testing the loss of both *Hyg* and *int* genes. A 579-bp fragment was expected from amplification of the *Hyg* gene open reading frame with Hyg0702-f and Hyg0702-r; a 586-bp fragment from *int* with Int0702-f and Int0702-r; and a 367-bp fragment from the genome of MTB::pOPHI with deleted *dif*-ΩHYG-*int*-*dif* using primers noHI-f and noHI-r. Three randomly selected MTB H37Rv::pOHIhd colonies with lost HYG resistance gene were amplified with primers attB1210f and attB1210r (Table 3) and sequenced to verify if the whole pOHIhd (Table 2) plasmid had been lost in these strains. Three MSM colonies co-transformed with pInt and pOHP (Table 2) were verified further by amplification with primers luxAB-f and luxAB-r (Table 3), and the expected PCR product was 750bp. The bioluminescence of the autoluminescent MSM/BCG/MTB H37Ra transformants was detected by GloMax 20/20 Luminometer (Promega) while for the autoluminescent MTB H37Rv transformants was detected by Orion II Microplate Luminometer (Titertek-Berthold).

### Testing the stability of the selectable marker-free autoluminescent MSM, BCG and MTB

Three single colonies of selectable marker-free autoluminescent MSM, BCG and MTB H37Rv were separately inoculated into 30 mL 7H9 medium and incubated at 37°C with shaking until the OD_600_ reached over 0.7. An aliquot of 0.3 ml of the culture was then sub-cultured into 30 mL 7H9 medium under the same conditions. An appropriate dilution of the broth culture was obtained after several passages and then plated on plain 7H11 plates. The RLUs of approximately 200 individual colonies picked up at each time point was detected using the above mentioned luminometers. The proportion of autoluminescent colonies was then calculated as: the No. of positive colonies/the total number of colonies detected×100%. If >99% colonies were still autoluminescent after 3 passages (~20 generations), this indicated that the strain was very stable.

## Results

### Construction of marker-free autoluminescent mycobacteria

We endeavored to create selectable marker-free mycobacteria by removing the resistance marker using the exogenous resolvase or the endogenous mycobacterial recombinases XerCD. In the first strategy, target strains were to be created by integrating the *Hsp60-luxCDABE* and the *res*-ΩKAN-*res* cassette containing plasmids into the genomic *attB* site with *int* gene in a separate plasmid. This was to be followed by the removal of the KAN resistance maker gene by the tnpR from resolvase of transposon γδ system, and subsequent removal of the plasmid expressing the resolvase [22]. Even though this *tnp*/*res* system had been proved successful in MSM [22], it was unsuccessful in this study using autoluminescent MSM and therefore we did not proceed with it using MTB.

On the other hand, we succeeded using the second strategy in which the target selectable marker-free autoluminescent strains were constructed by integrating the *Hsp60-luxCDABE* into the genome, followed by the removal of the resistance gene together with the *int* gene by the endogenous recombinases XerC and XerD [14].

Both MSM and MTB H37Rv were transformed with pOHIhd or pOPHI successfully (Fig. 1, Table 2). Thereafter, BCG and MTB H37Ra were also transformed with pOPHI successfully. All transformants colonies were verified further by detecting bioluminescence. We co-transformed pInt and pOHP (Fig. 1, Table 2) into MSM successfully and obtained MSM-OHP (Table 1). However, none of them was bioluminescent. We therefore verified by PCR if the *luxCDABE* and *Hyg* fragments had been integrated into the MSM genome using primer pairs luxAB-f and luxAB-r (750-bp band), and Hyg0702-f and Hyg0702-r (579-bp band), respectively (Table 3). All the 3 randomly selected MSM-OHP colonies gave right sized bands, which meant that the plasmid pOHP (Table 2) had been integrated into the MSM genome.

### Counter-selection of the selectable marker-free autoluminescent mycobacteria

The selectable marker-free autoluminescent mycobacterial colonies with HYG-resistant gene rescued were screened by passing the corresponding parent strains several times in antibiotic-free broth culture, testing HYG susceptibility and the autoluminescence of each individual colony. For MSM transformants containing pOHIhd (Fig. 1, Table 2) and designated as AlMSMT1 (Table 1), 90% colonies did not grow on HYG-containing plates anymore after just one passage, and had also lost their autoluminescence. No selectable marker-free autoluminescent MSM was obtained through this technique route after multiple attempts. A similar phenomenon was observed in MTB H37Rv strain transformed with the same plasmid and designated as AlRvT1 (Table 1).

We suspected that the plasmid pOHIhd could have been dissociated from the genome of AlMSMT1 or AlRvT1 much faster than the dissociation of *dif*-ΩHYG-*dif* cassette by the endogenous XerCD. Therefore primers attB1210-f and attB1210-r (Table 3) were designed for amplification of the 700-bp fragment containing *attB* in the middle. Genomic DNA from three randomly selected AlRvT1 colonies without bioluminescence was used as templates with that of wild-type H37Rv as the control. All gave a ~700-bp fragment (S4 Fig.), and the sequence of the randomly selected PCR product from lane 3 was the same as that of wild-type H37Rv.

**Fig 2:**
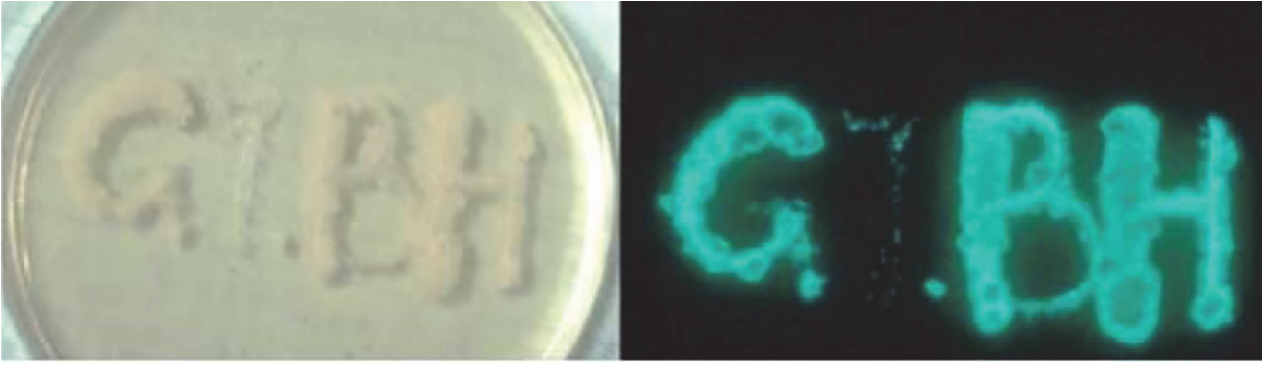
Photograph of the more stable, selectable marker-free, autoluminescent BCG (UABCG).

MSM transformed with pOPHI and designated as AlMSMT2 (Table 1) was passed twice in drug-free 7H9 broth and plated on plain agar. 56% colonies lost their HYG resistance and were still autoluminescent and one representative strain was designated as UAlMSM (Table 1). MTB H37Rv transformed with pOPHI was designated as AlRvT2 (Table 2), and all 200 AlRvT2 colonies had lost the HYG resistance and were also still autoluminescent after just one passage. One representative selectable marker-free autoluminescent MTB H37Rv strain was designated as UAlRv (Table 1). Similarly, we obtained AlRaT2 and AlBCGT2 by transforming MTB H37Ra and BCG respectively with pOPHI (Table 2) and the corresponding selectable marker-free autoluminescent UAlRa and UABCG (Fig. 1, Table 1). Two randomly selected UAlMSM, UABCG (Fig. 2), UAlRa and UAlRv colonies (Table 1) were verified further for the loss of the *Hyg* and *int* genes by PCR using 3 primer pairs (Table 3): Hyg0702-f and Hyg0702-r for detecting loss of *Hyg*, Int0702-f and Int0702-r for detecting loss of *int*, and noHI-f and noHI-r for detecting the loss of both *Hyg* and *int* genes. As expected, no right sized PCR products were obtained using the first 2 primer pairs and a 367-bp fragment was obtained using the primers noHI-f and noHI-r (S5 Fig.). Sequence analysis showed that the randomly selected target band from UAlRv1 (lane 2) was the same as deduced from AlRvT2 genome with the *dif-ΩHYG-int-dif* cassette lost (Table 1). At last, we obtained the selectable marker-free autoluminescent MSM, BCG and MTB H37Rv (Table 1) with *int* gene lost using pOPHI. The selectable marker-free autoluminescent mycobacterial colonies were visible with naked eyes in a dark room and could be imaged using a normal camera.

### Stability of the selectable marker-free autoluminescent mycobacteria

The stability of UAlMSM, UAlRa, UAlRv and UABCG was tested. For UAlMSM, >99% colonies were still autoluminescent after 6 passage (~40 generations) in about 4 weeks, which implied that the strain was very stable. About 100% (at least more than 99%) of the UAlRv and UAIRa colonies were still strongly autoluminescent after 2 (about 1 month, (~15 generations) and 5 (about 94 days, >35 generations) passages respectively. The UABCG was also very stable as it retained autoluminescence after several passages within 3 months.

## Discussion

Studies in MTB and other mycobacteria, and especially the discovery of new anti-mycobacterial drugs and the mechanism of drug action are heavily hampered by their slow growth and the need of expensive biosafety laboratory at higher levels. Rapid, convenient, inexpensive and sensitive reporter strains would facilitate such studies in mycobacteria. We previously demonstrated that a very sensitive autoluminescent MTB grew as fast and was as virulent as its parent strain, in which the RLUs produced by this strain accurately correlated with the CFU counts. The strain could not only be used *in vitro* for rapid evaluation but also *in vivo* for rapid drug and even vaccine testing noninvasively using the same batch of live mice in a larger scale [7]. However, there were 2 deficiencies in the autoluminescent strain. Firstly, the strain was not stable, which would limit its applications, and secondly, it contained a KAN resistance marker gene, which further limited its utility in many fields, especially for MTB which has only *Hyg* and KAN resistance markers [12].

In this study, we demonstrate for the first time the successful construction of selectable marker-free autoluminescent mycobacteria including MTB, BCG and MSM (Table 1). More importantly, all the target strains were extremely stable. For example, 100% (at least >99%) of randomly selected individual colonies of the UAlRv were still autoluminescent after 5 passages with >35 generations in 94 days comprising both log phase and stationary phase culture. In contrast, only 95.7% colonies of the KAN-resistant autoluminescent AlRv (Table 1) were autoluminescent after 37 days of *in vitro* growth in broth without passage [7]. These results were accordant with a previous study describing the construction of recombinant MTB expressing firefly luciferase gene in which the strains whose *int* gene was removed were more stable than those whose *int* gene was not removed [11]. One limitation of this study is that we did not test stability under diverse growth conditions, such as, low pH, macrophage infection model, non-replicating persistence as well as infection animal models. The macrophage infection model can not last for a very long time (usually within 14 days), and no loss of bioluminescence because of instability was observed in our study thus indicating sufficient stability of our strain in the model. The integration of the transforming plasmid into the genomes of mycobacteria and subsequent removal of the integrase gene which excises the plasmid at a very low rate contributed to the stability of the target strains in this study. However, further stability testing of such mycobacterial strains under the above diverse conditions would be needful.

The *dif-ΩHYG-int-dif* cassette could be widely used in constructing selectable marker-free and more stable recombinant MTB or BCG strains in just one transformation step, such as BCG-based vaccines and recombinant MTB reporter strains.

Previously, no autoluminescent mycobacteria were successfully constructed using extrachromosomal plasmids as delivery vectors [7]. Whether this arose from the reaction of bioluminescence triggering some unknown mechanism to eliminate the plasmids is unknown. Additionally, whether the extra-chromosomal plasmids are affected by the luminescence produced in the autoluminescent mycobacteria challenging their stable existence is also unknown. Another possible cause of instability of the extrachromosomal plasmids expressing *luxCDABE* in recombinant mycobacteria is the lack of enough energy and toxicity arising from the strong autoluminescence reaction [15]. An earlier study reported that the GFP is expressed at a much higher level when its gene is carried in an extrachromosomal plasmid than when carried in an integrative plasmid [23]. We also reported in our previous study that if a strong promoter is in front of *luxAB*, such a plasmid could not be obtained even in *E*. *coli* because of high toxicity [15]. In this study, we also transformed several types of extrachromosomal plasmids into the selectable marker free autoluminescent mycobacteria and found they could stably exist in them (data not shown). The findings of this study and the other two studies mentioned above support the latter hypothesis about the instability of extrachromosomal plasmids expressing *luxCDABE*. However, the real reason for this phenomenon still needs further verification as such extrachromosomal plasmids could be used in autoluminescent mycobacteria for studying mechanisms of drug action, such as over-expression and gene complementary experiments.

In a previous study, the authors reported the inability to recover the wild-type *attB* sequence of MSM in *E*. *coli* acceptor cells due to consistent rearrangements [24]. They hypothesized that the wild-type *attB* sequence of MSM is toxic to *E*. *coli* due to the presence of the mycobacterial tRNAgly within the *attB* site. However, using our failed strategy, we obtained the wild-type *attB* site-containing plasmid from *E*. *coli* without any problem in this study. One difference between these 2 studies is the type of *E*. *coli* strains used, as they had used *E*. *coli* strain SH288 while we used *E*. *coli* DH5α. The other difference is that the fragment containing *attB* site in our study contained an intact tRNAgly, while in the reported study it only contained a partial tRNAgly [24], which could have been toxic to *E*. *coli*.

One interesting observation is that when the mycobacteria were transformed with pOHIhd (Fig. 1, Table 2) in which the *Hyg* was supposed to be removed by the XerCD, the transformants were autoluminescent. However, all the selectable marker-free derivatives could not give out light. When checked, the selectable marker-free colonies had lost the pOHIhd plasmid at the *attB* site and were recovered as wild-type. Therefore, selectable marker-free autoluminescent mycobacteria strains could not be obtained by this method. This phenomenon was however not observed with pOPHI (Fig. 1, Table 2) in which the *int* and *Hyg* genes were lost together. The autoluminescence together with *dif* sequence (XerCD) could have affected the activity of the integrase in pOHIhd. Besides, we did not encounter a similar phenomenon in our previous study using eGFP contained in the integrative plasmid pTYGi9 instead of the *luxCDABE* [12]. The mycobacteria strains transformed with pTYGi9 can just lose the *dif*-ΩHYG-*dif* cassette alone successfully instead of the whole plasmid. However, the exact reason for the above strange phenomenon is not fully established and still needs to be further investigated.

The selectable marker-free and more stable mycobacteria present several obvious advantages: Firstly, there is no need of regrowing the original autoluminescent MTB very often to avoid loss of bioluminescence during drug screening and evaluation. Secondly, the potential cross-resistance during drug screening arising from the *Kan* gene is eliminated. Additionally, the strains can be used to test regimens containing KAN or any drug combinations with KAN. Thirdly the selectable marker-free autoluminescent mycobacterial strains can be used for mycobacterial recombineering [25] or for creating unmarked deletions in autoluminescent strains [26]. Mycobacterial recombineering is a very useful tool that was recently developed for knocking out mycobacterial gene(s) [25], and requires mycobacteria containing a plasmid expressing the *gp60/61* genes for increasing the recombination rate. The substrate for homologous exchange usually contains another resistance marker, and as mentioned above, only *Kan* and *Hyg* marker genes are utilized in MTB which means that the parent strain should be selectable marker-free. Fourthly, the strains created here can be used for high efficient transposition experiments directly. The mycobacteriophage carrying the highly efficient transposon harbor a *Kan* marker gene, and when used to transpose MTB [27], the subsequent complementary experiments require the use of another drug resistant marker. Fifthly, some clinical isolates could already be resistant to KAN, and thus after transformation of such a KAN-resistant strain with a *Hyg* gene to make it autoluminescent; it would be very hard to do any further transformation. Sixthly, the strains have the potential to study the mechanism of drug action related genes more efficiently and quickly. For example, in the knocking out of a gene and complementing it with the corresponding mutant; or overexpressing a gene in the selectable marker-free stable autoluminescent mycobacteria; and then testing their susceptibilities to the corresponding drugs. According to our previous published data on the resistance gene marked autoluminescent strains, it is very reasonable to infer that the new version of strains are more suitable for anti-mycobacterial drug research and for studing the functions or virulence of genes rapidly and more intuitively.

In summary, there existed some unresolved problems associated with autoluminescence, stability and the integration system of L5 mycobacteriophage in mycobacteria. In this study, however, we have successfully created selectable marker-free autoluminescent mycobacteria by just one transformation which presents many advantages than the previous versions.

## Supporting Information

S1 Fig Construction of the plasmid pOHIhd. *oriE*, origin region of *E*. *coli*; *bla*, ampicillin resistance gene; *Hyg*, HYG resistance gene; *dif*, the recombinases XerCD action site; *Hsp60*, the strong mycobacterial promoter; *luxCDABE*, the operon for producing autoluminescence; *attP*, mycobacteriophage L5 attachment site; *int*, integrase gene. Commonly used restriction enzyme sites are indicated.
(TIF)

S2 Fig Construction of the plasmid pOPHI. *oriE*, origin region of *E*. *coli*; *bla*, ampicillin resistance gene; lacZ, the beta-galactosidase gene; lacZ’ and lacZ”, the remaining parts of beta-galactosidase gene; *dif*, the recombinases XerCD action site; *int*, integrase gene; *int’*, the remaining part of integrase gene; *Hyg*, HYG resistance gene; *Hsp60*, the strong mycobacterial promoter; *luxCDABE*, the operon for producing autoluminescence.
(TIF)

S3 Fig Construction of the plasmid pTYOP. *oriE*, origin region of *E*. *coli*; *bla*, ampicillin resistance gene; *attP*, mycobacteriophage L5 attachment site; *int*, integrase gene; *int’*, the remaining part of integrase gene; *Hsp60*, the strong mycobacterial promoter; *luxCDABE*, the operon for producing autoluminescence was from plasmid pluxOK. Commonly used restriction enzyme sites are indicated.
(TIF)

S4 Fig Identification of AlRvT1. MTB H37Rv transformed the pOHIhd colonies that lost the autoluminescnece by PCR with primers attB1210-f and attB1210-r. M, DNA marker; 1, wild type MTB H37Rv as a control; 2–4, three randomly selected AlRvT1 colonies from that lost the autoluminescnece.
(TIF)

S5 Fig Identification of dif-ΩHYG-int-dif deletion in UAlRv, UABCG and UAlMSM using primers noHI-f and noHI-r. Lane M, DNA marker (bp); Lane 1, PCR product from water as a control (no template); Lane 2,3, PCR products from UAlRv colony 1 and colony2; Lane 4,5, PCR products from UABCG colony 1 and colony2; Lane 6,7, PCR products from UAlMSM colony 1 and colony2; Lane 8, product from wild-type BCG as a control. The right band from lane 2 was sequenced.
(TIF)
