# Supplementary figures and images for: Engineering More Stable, Selectable Marker-Free Autoluminescent Mycobacteria by One Step
Source: PLoS One. 2015 Mar 11;10(3):e0119341. doi: 10.1371/journal.pone.0119341 (PMC4356594; doi:10.1371/journal.pone.0119341)

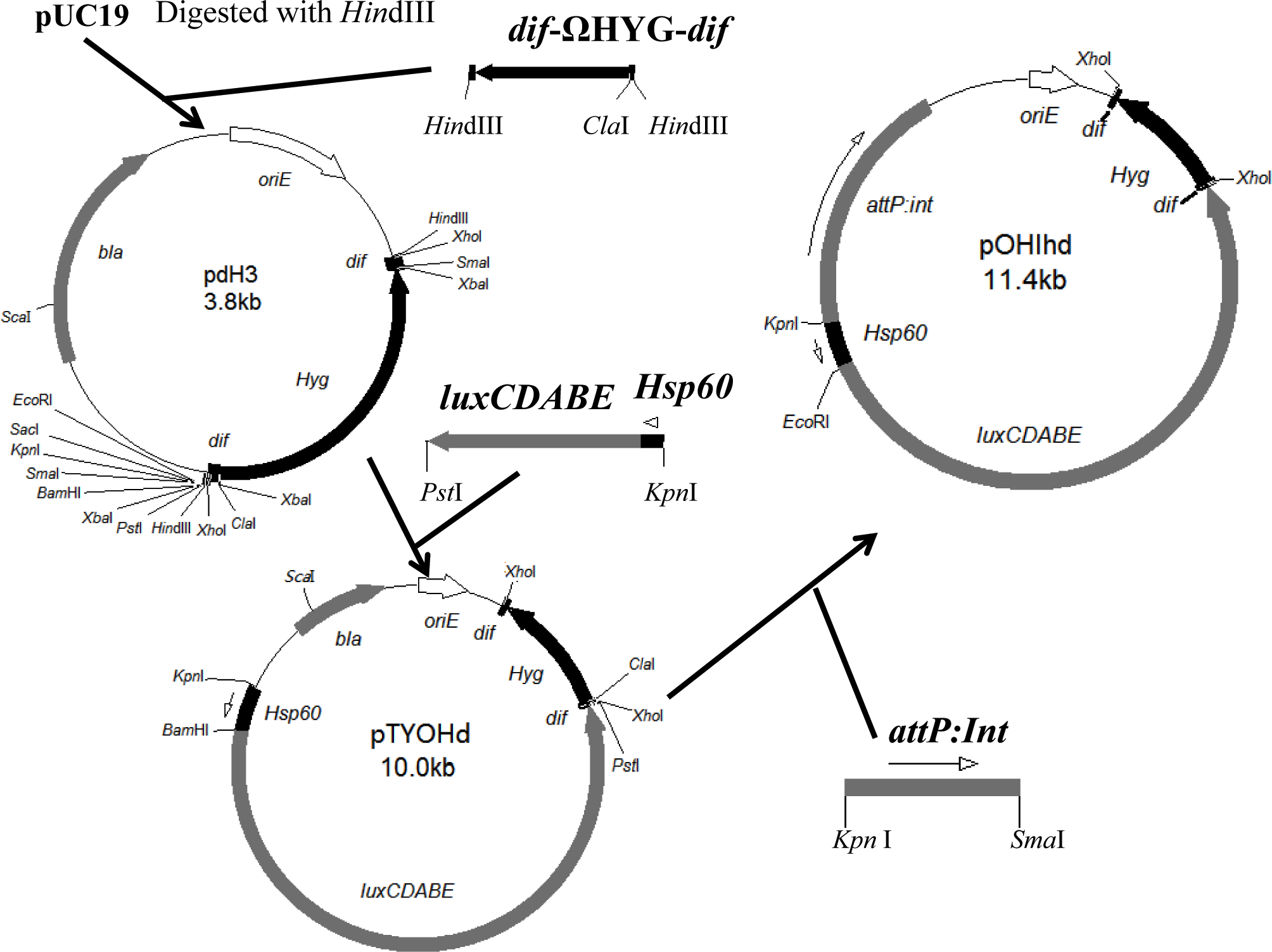

Supplement: S1 Fig — oriE, origin region of E. coli; bla, ampicillin resistance gene; Hyg, HYG resistance gene; dif, the recombinases XerCD action site; Hsp60, the strong mycobacterial promoter; luxCDABE, the operon for producing autoluminescence; attP, mycobacteriophage L5 attachment site; int, integrase gene. Commonly used restriction enzyme sites are indicated. (TIF) [file pone.0119341.s001.tif]

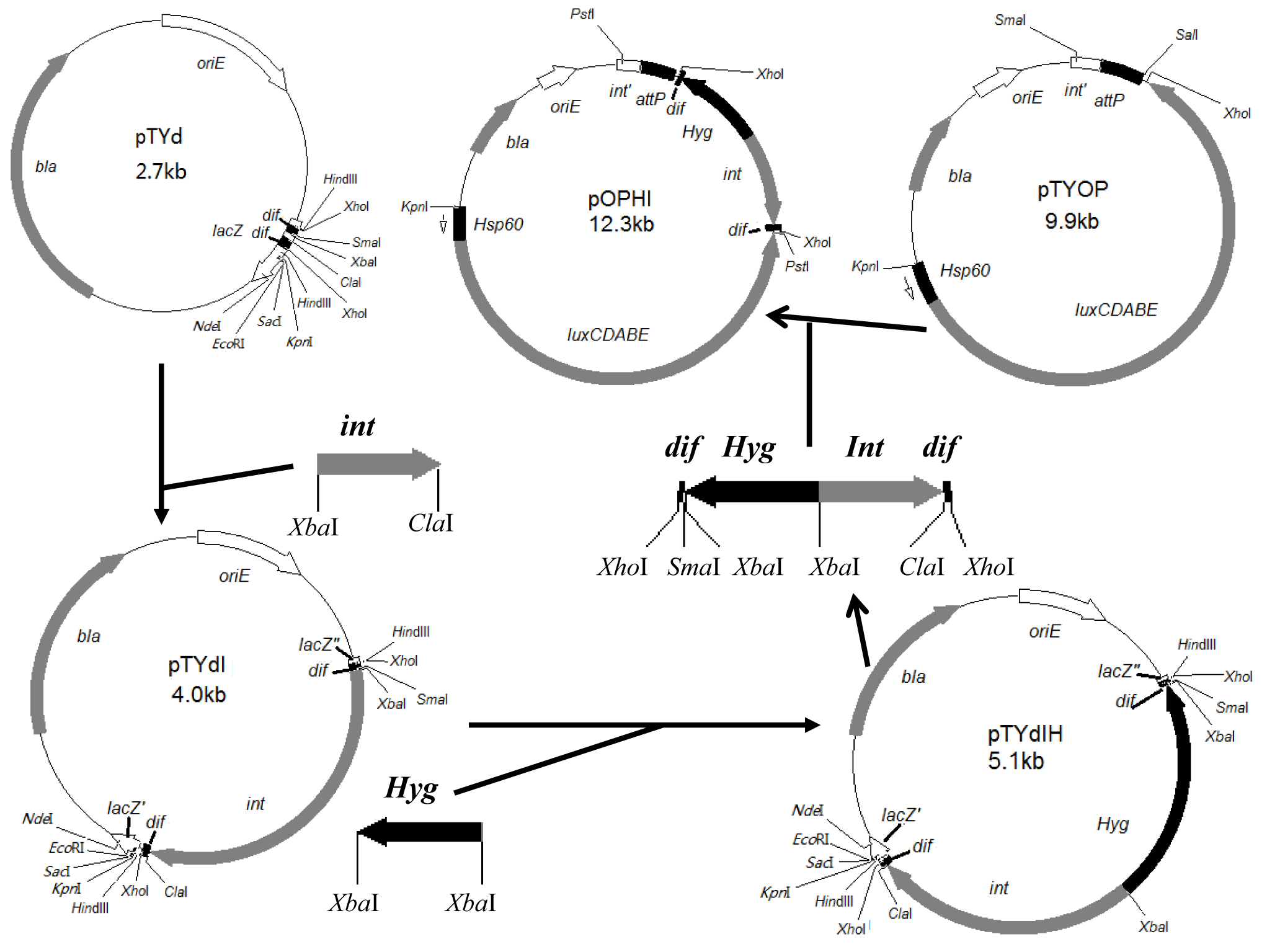

Supplement: S2 Fig — oriE, origin region of E. coli; bla, ampicillin resistance gene; lacZ, the beta-galactosidase gene; lacZ’ and lacZ”, the remaining parts of beta-galactosidase gene; dif, the recombinases XerCD action site; int, integrase gene; int’, the remaining part of integrase gene; Hyg, HYG resistance gene; Hsp60, the strong mycobacterial promoter; luxCDABE, the operon for producing autoluminescence. (TIF) [file pone.0119341.s002.tif]

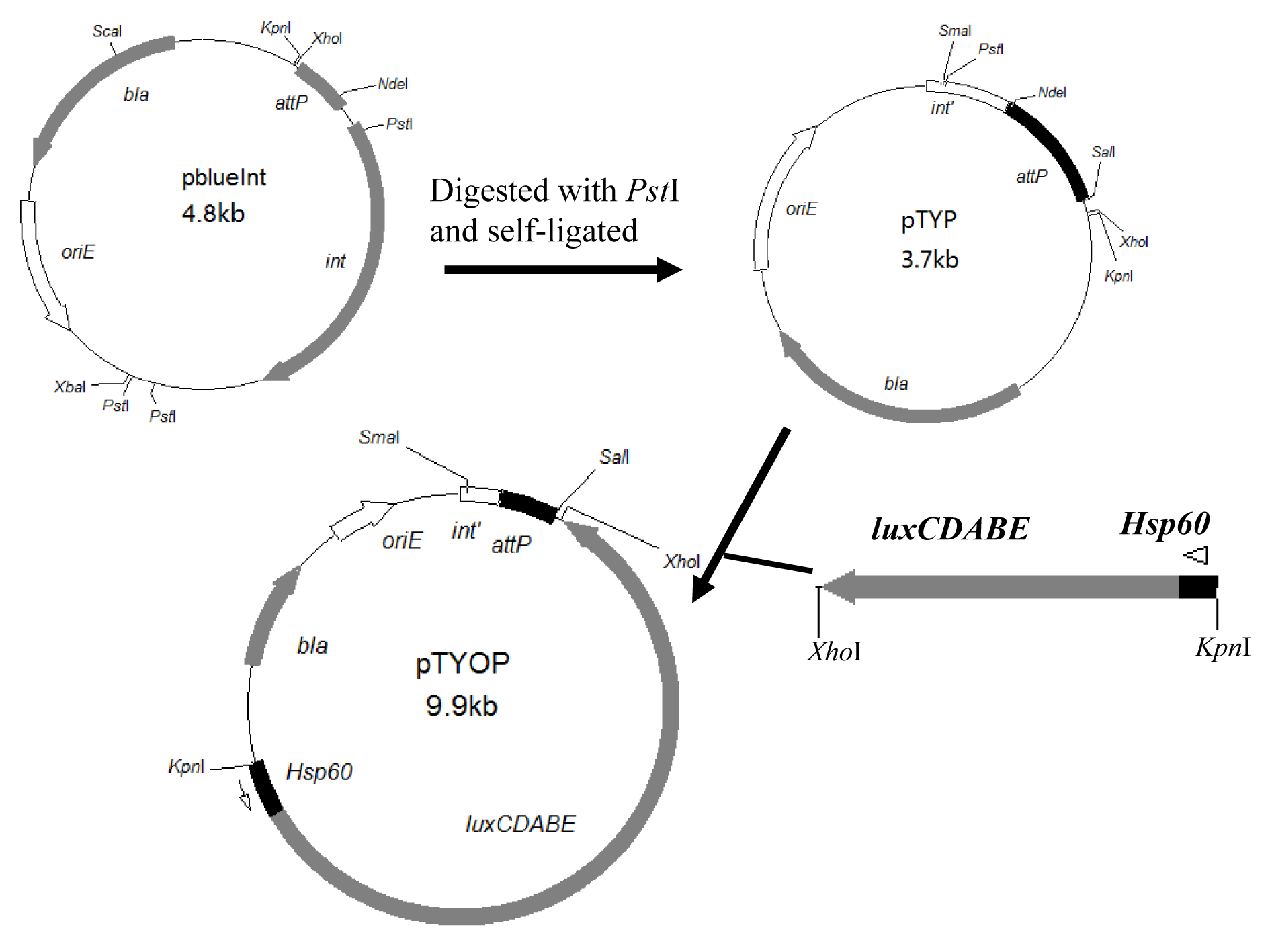

Supplement: S3 Fig — oriE, origin region of E. coli; bla, ampicillin resistance gene; attP, mycobacteriophage L5 attachment site; int, integrase gene; int’, the remaining part of integrase gene; Hsp60, the strong mycobacterial promoter; luxCDABE, the operon for producing autoluminescence was from plasmid pluxOK. Commonly used restriction enzyme sites are indicated. (TIF) [file pone.0119341.s003.tif]

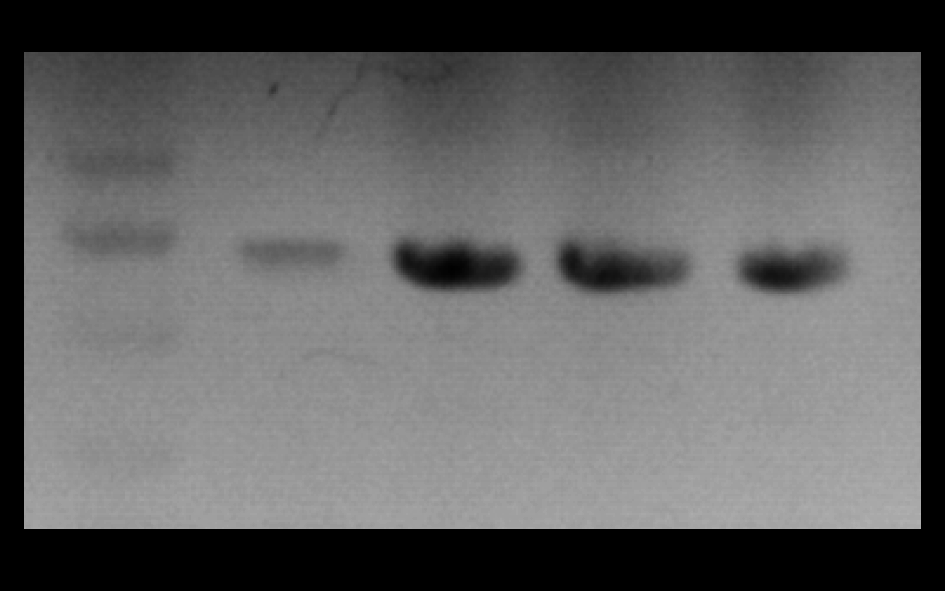

Supplement: S4 Fig — MTB H37Rv transformed the pOHIhd colonies that lost the autoluminescnece by PCR with primers attB1210-f and attB1210-r. M, DNA marker; 1, wild type MTB H37Rv as a control; 2–4, three randomly selected AlRvT1 colonies from that lost the autoluminescnece. (TIF) [file pone.0119341.s004.tif]

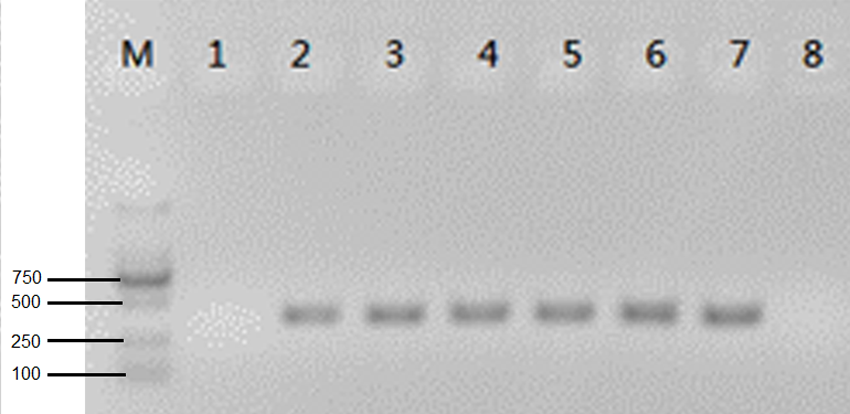

Supplement: S5 Fig — Lane M, DNA marker (bp); Lane 1, PCR product from water as a control (no template); Lane 2,3, PCR products from UAlRv colony 1 and colony2; Lane 4,5, PCR products from UABCG colony 1 and colony2; Lane 6,7, PCR products from UAlMSM colony 1 and colony2; Lane 8, product from wild-type BCG as a control. The right band from lane 2 was sequenced. (TIF) [file pone.0119341.s005.tif]
